# Supplementary figures and images for: Analysis of Spatio-Temporal Transcriptome Profiles of Soybean (Glycine max) Tissues during Early Seed Development
Source: Int J Mol Sci. 2020 Oct 14;21(20):7603. doi: 10.3390/ijms21207603 (PMC7589660; doi:10.3390/ijms21207603)

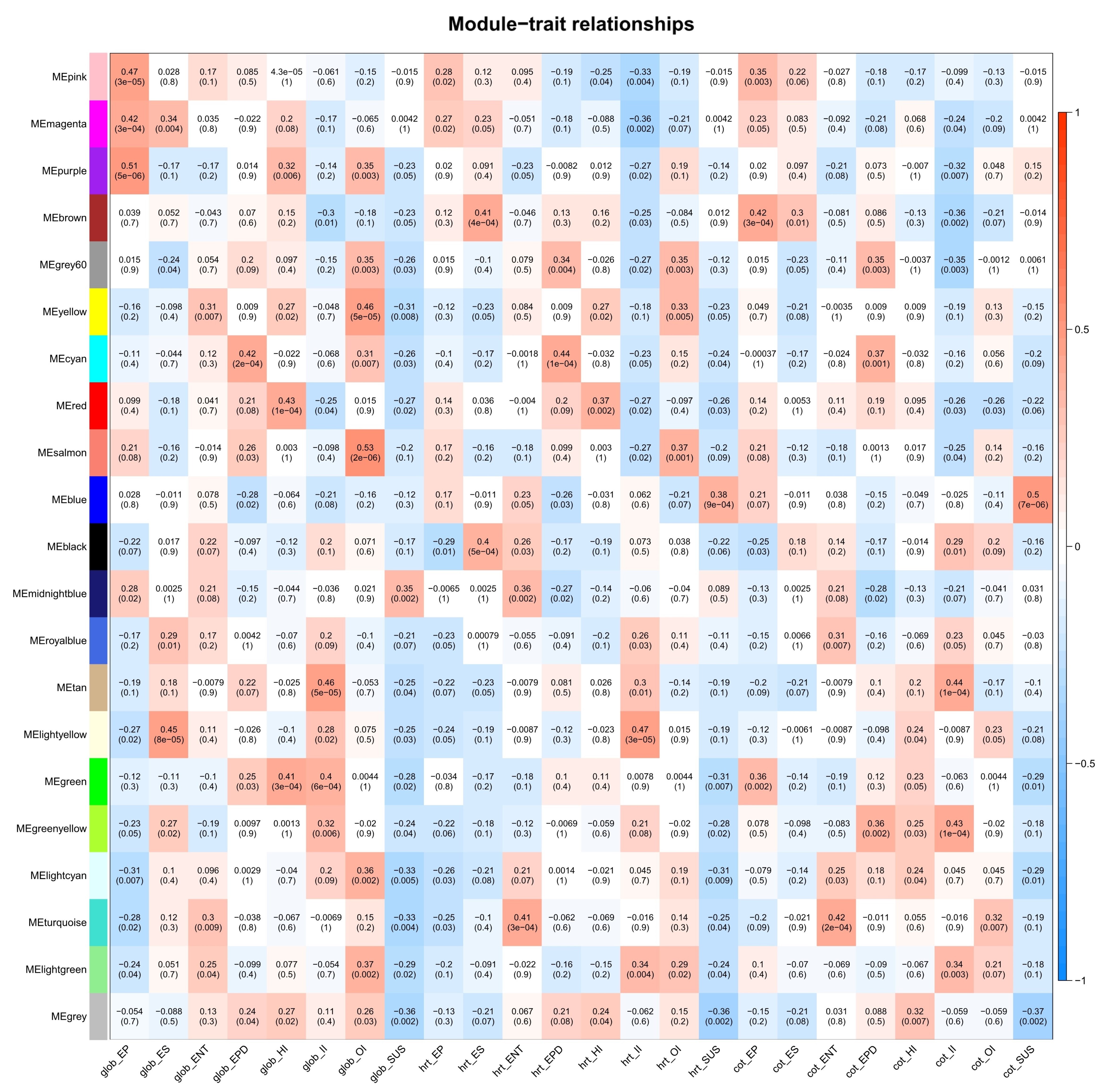

Supplement: Supplementary file 1 [file ijms-21-07603-s001.zip › supplementary_files/Figure S1.jpg]
